# Correction: Cytosolic PCNA interacts with p47phox and controls NADPH oxidase NOX2 activation in neutrophils

**DOI:** 10.1084/jem.2018037111082019c

**Published:** 2019-11-15

**Authors:** Delphine Ohayon, Alessia De Chiara, Pham My-Chan Dang, Nathalie Thieblemont, Simon Chatfield, Viviana Marzaioli, Sabrina Sofia Burgener, Julie Mocek, Céline Candalh, Coralie Pintard, Pascale Tacnet-Delorme, Gilles Renault, Isabelle Lagoutte, Maryline Favier, Francine Walker, Margarita Hurtado-Nedelec, Dominique Desplancq, Etienne Weiss, Charaf Benarafa, Dominique Housset, Jean-Claude Marie, Philippe Frachet, Jamel El-Benna, Véronique Witko-Sarsat

Vol. 216, No. 11, November 4, 2019. 10.1084/jem.20180371.

The authors regret that in their original paper, the TNBS+anti-Ly6G+T2AA image in Fig. 8 D was incorrect as a result of an error during figure preparation. In addition, y axes and histograms were missing in panels B and E due to a production error. The corrected figure appears below.

**Figure 8.**
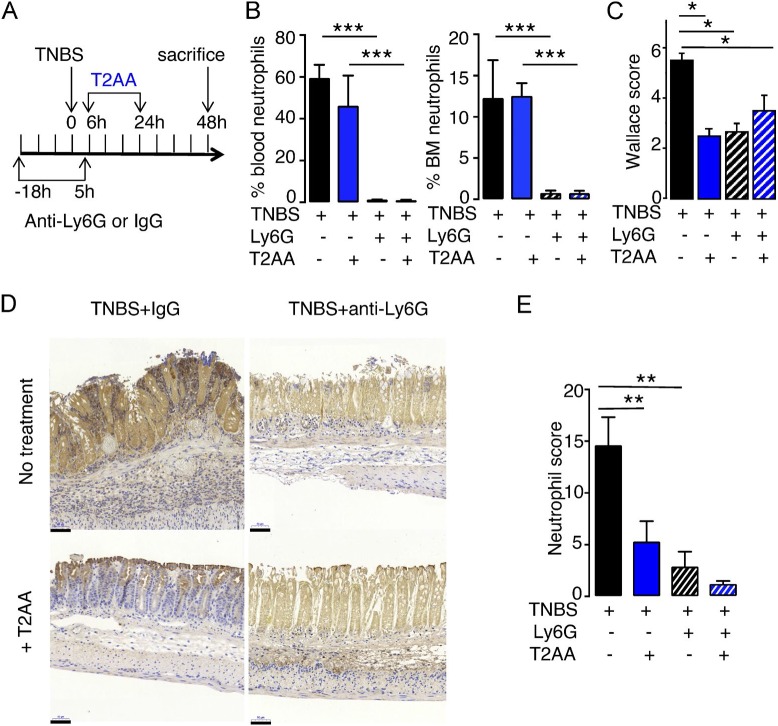
**Absence of T2AA antiinflammatory effect in TNBS-induced colitis following neutrophil depletion. (A)** Experimental protocol of colitis after neutrophil depletion using anti-Ly6G mAb. **(B and C)** Flow cytometry quantification of neutrophils in blood (left panel) and in bone marrow (right panel) in control (IgG) and neutrophil-depleted (Ly6G) mice. (C) Macroscopic changes following TNBS treatment measured by Wallace score. The data are mean ± SEM (ANOVA). **(D)** Representative photomicrographs of neutrophil immunostaining of colonic tissues with the neutrophil marker recovered from control (IgG) and neutrophil-depleted mice (Ly6G) treated with TNBS alone or TNBS combined with T2AA. The scale bars correspond to 20 µm, magnification 200×. **(E)** Quantification of neutrophil immunostaining as described in Materials and methods. In B, C, and E, the data are mean ± SEM. Two independent sets of experiments were performed with a total of eight mice per group. *, P < 0.05; **, P < 0.01; ***, P < 0.001, ANOVA.

The online HTML and PDF have been corrected. The error remains only in the print version.

